# Unraveling a Hidden Mass: A 10‐Year Mystery of Groin Swelling Reveals an Unexpected Diagnosis of Neurofibroma

**DOI:** 10.1002/ccr3.72246

**Published:** 2026-03-11

**Authors:** Alaa Tajeldeen Habeeb Abdallah, Zainab Hassan Nooreldayem, Rami Elsiddig Abdelkhalig, Elwasila Hamid, Nouh Saad Mohamed, Ayman Ahmed, Emmanuel Edwar Siddig

**Affiliations:** ^1^ Faculty of Medicine University of Khartoum Khartoum Sudan; ^2^ Rufa'a Teaching Hospital Albutana University Rufaa Sudan; ^3^ Molecular Biology Unit Sirius Training and Research Centre Khartoum Sudan; ^4^ Institute of Endemic Diseases University of Khartoum Khartoum Sudan; ^5^ Pan‐Africa One Health Institute (PAOHI), Kigali 11KG ST203 Kigali Rwanda; ^6^ Rwanda Biomedical Center (RBC) Kigali Rwanda; ^7^ Faculty of Medical Laboratory Sciences University of Khartoum Khartoum Sudan

**Keywords:** diabetes mellitus, groin swelling, histopathology, neurofibroma, surgery, umbilical hernia

## Abstract

This case presents the perplexing journey of a 70‐year‐old woman with a decade‐long history of painless, gradually enlarging left‐side groin swelling. Despite initial suspicion of a hernia, the surgical exploration uncovered an unexpected finding: a well‐circumscribed oval mass. Histopathological analysis led to the final diagnosis of neurofibroma. This case sheds light on the importance of thorough investigation in puzzling presentations and provides valuable insights into the clinical manifestations, differential diagnosis, investigations, and management of neurofibromas.

## Key Clinical Message

Groin neurofibromas present a diagnostic challenge as they can mimic hernias, requiring a systematic approach involving thorough history‐taking, careful physical examination, appropriate imaging techniques, and definitive histopathological analysis to ensure accurate diagnosis and effective management.

## Graphical abstract

Unravelling a Hidden Mass: Case of 70‐year‐old female presenting with 10‐year history of painless groin swelling. Intraoperative discovery revealed an unexpected neurofibroma.

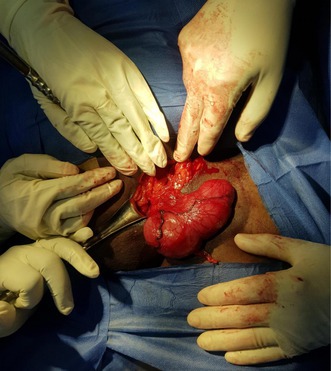

## 1 Introduction

Neurofibromas are benign tumors that originate from peripheral nerves [1]. They can occur sporadically, meaning they develop without any underlying genetic condition, or they can be associated with a genetic disorder called neurofibromatosis [1, 2]. Neurofibromatosis can be further classified into two types: neurofibromatosis type 1 (NF1) and neurofibromatosis type 2 (NF2) [1, 2, 3].

Neurofibromas can develop in various parts of the body, with the skin, soft tissues, and peripheral nerves being the most common sites. They typically present as painless, slow‐growing masses that are soft and well‐defined [3, 4].

While the prevalence of solitary neurofibromas is not as well‐documented as neurofibromatosis Type 1 (NF1), the reported incidence of NF1 is approximately 1 in 3000 individuals worldwide [5]. Solitary neurofibromas manifest as isolated tumors, contrasting with NF1, which is characterized by multiple neurofibromas, café‐au‐lait spots, plexiform neurofibromas, and various systemic symptoms [3, 5, 6, 7]. The differential diagnosis for solitary neurofibromas includes schwannoma, lipoma, hernia, fibroma, vascular malformations, dermatofibromas, and other soft tissue tumors [3, 8]. In this report, we present a case of a neurofibroma that was initially suspected to be a hernia.

## 2 Case Presentation

A 70‐year‐old woman presented with a long‐standing left‐side groin swelling. The swelling had gradually increased in size over a 10‐year period, following a uniform growth pattern. It was painless, limitedly mobile in the inguino‐labial region, and more prominent in the upright position. The swelling did not change in size during coughing or straining; however, it caused her discomfort and a sensation of heaviness. The patient initially believed it to be a hernia, similar to the umbilical hernia she had for the past 3 years.

Her medical history is significant for type 2 diabetes mellitus, diagnosed 5 years ago, and hepatitis B infection, diagnosed 3 years ago. The patient has had nine pregnancies, with eight delivered vaginally and the last by cesarean section in 2004. Other than these conditions, her past medical and family histories were unremarkable.

Upon general physical examination, the patient was not pale or jaundiced. A close examination revealed a left inguino‐labial swelling. The swelling was oval in shape, with well‐defined margins, and measured approximately 10 × 6 cm. It was soft in consistency, non‐tender, irreducible, and limitedly mobile. There was no attachment to the skin, which appeared normal overlying the area. The temperature of the skin was normal compared to the surrounding tissue. Additionally, a concomitant 6 × 6 cm umbilical swelling was observed; this swelling was soft, reducible, and showed a positive cough impulse. No other swellings were noted, and there were no café‐au‐lait spots or signs indicative of neurofibromatosis. The remainder of the systemic examination was unremarkable.

## 3 Methods

**FIGURE 1:**
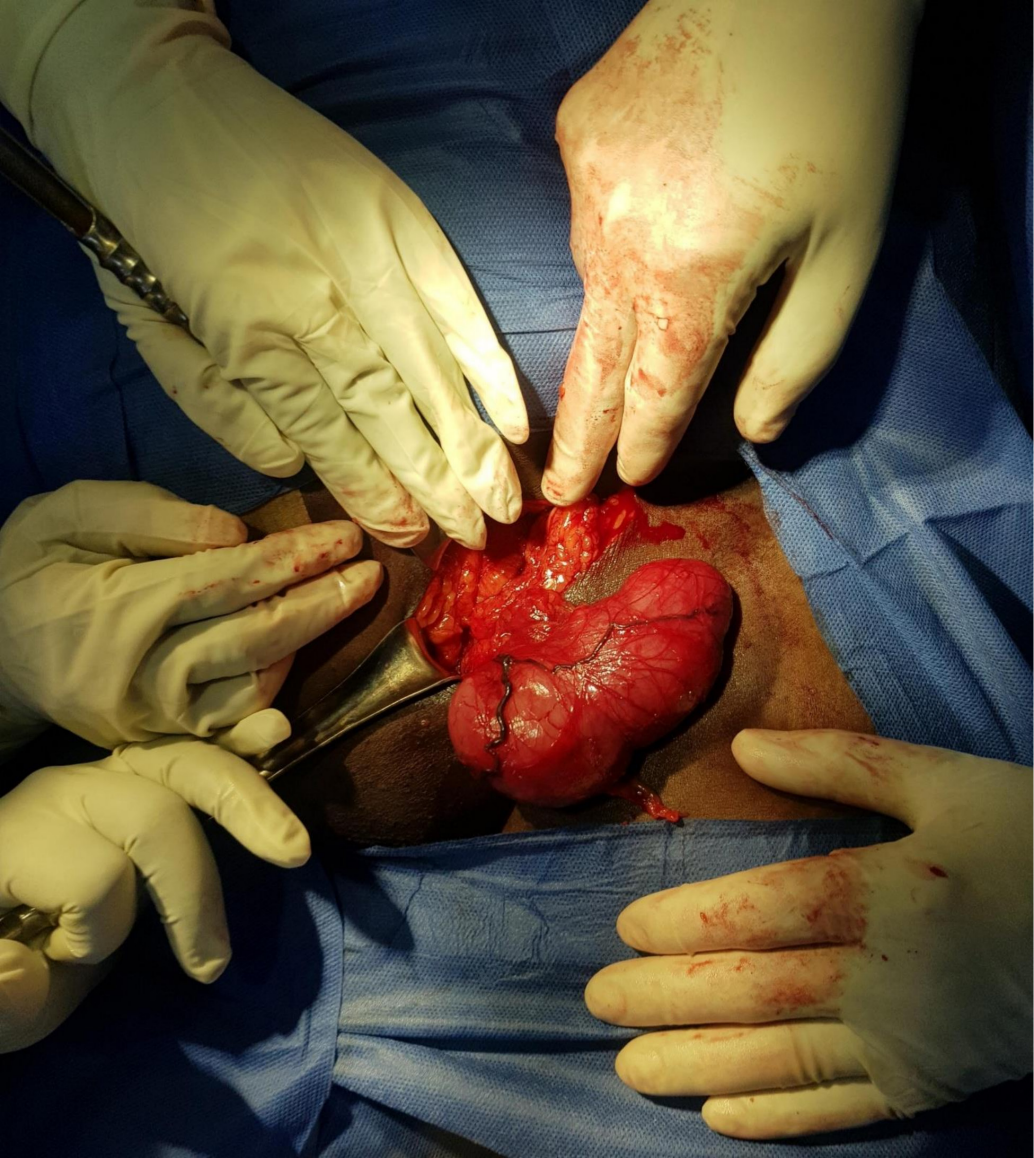
Over the left inguinal region, a surgical incision was made, and the abdominal layers were dissected and retracted, revealing a well‐circumscribed mass. The mass was found to be a neurofibroma.

Initially, the working diagnosis for the painless, soft groin swelling was hernia. An open hernia repair operation under spinal anesthesia was planned, aiming to ascertain the presence of an inguinal or femoral hernia. However, upon incision, no hernia or defect was found. Instead, a distinct whitish‐to‐grayish oval mass was discovered (Figure 1). The mass was sent for histopathological analysis. The cross section showed a firm tan and whitish whorly surface, and the microscopic examination of the mass revealed the proliferation of all elements of peripheral nerves, including Schwann cells with wire‐like collagen fibrils, stromal mucosubstances, Wagner–Meissner corpuscles, neurofilaments, fibroblasts, and collagen. Irregularly expanded nerve bundles with nodularity and myxoid areas were observed, with no evidence of atypia. The final diagnosis established was a neurofibroma. The patient fully recovered with no postoperative complications.

## 4 Conclusion and Results

One month postoperatively, an abdominopelvic ultrasound scan was performed, which confirmed the presence of an umbilical hernia containing fat, with the hernia orifice measuring approximately 1.6 cm. Other findings on the scan were unremarkable. She was followed for 2 years postoperatively, with no recurrence of the groin neurofibroma.

## 5 Discussion

This case represents a sporadic neurofibroma, as there were no café‐au‐lait spots or other clinical signs indicative of neurofibromatosis. Although solitary neurofibromas not associated with neurofibromatosis Type 1 (NF1) have been documented in patients aged 60 and older in regions such as the eyelid and epibulbar areas, our case is the first to document a solitary neurofibroma located in the groin area of an elderly patient [9, 10].

While specific prevalence statistics for neurofibromas are limited, insights from investigations in tertiary medical hospitals can provide valuable information. For instance, a decade‐long retrospective analysis in Nigeria identified 46 cases of neurofibromas, demonstrating a male‐to‐female ratio of 1.5:1, with peak incidence occurring in the fourth decade of life [11, 12]. Additionally, another study focused on surgical outcomes for benign peripheral nerve sheath tumors, including neurofibromas, reported a mean patient age of 39.4 years, with a notable predominance of females (14 females compared to 7 males) [13]. However, these studies do not distinguish between solitary neurofibromas and those associated with neurofibromatosis Type 1 (NF1), making it challenging to accurately ascertain the prevalence of solitary cases.

Neurofibromas, particularly those located in the abdominal wall and groin regions, can mimic hernias. An illustrative case involved a 45‐year‐old Nigerian female with an inguino‐labial neurofibroma that was initially diagnosed as a hernia [8]. Similarly, another case documented an abdominal wall neurofibroma presenting as an inguinal hernia in a young soldier [5]. More alarmingly, a report highlighted a neurofibrosarcoma misidentified as an inguinal hernia, emphasizing the serious consequences that can arise when neurogenic tumors are overlooked [14].

Neurofibromas are usually soft or firm, painless, slowly growing masses [3, 4]. Their presentation can mimic hernias, particularly in the groin, thereby increasing the risk of misdiagnosis. However, certain distinguishing features can aid in differentiating between the two: unlike hernias, neurofibromas are not reducible and tend to show specific characteristics, such as an oval shape and well‐defined margins [3, 8, 15].

Due to the commonality of groin hernias, initial clinical suspicion often gravitates toward hernia. Therefore, preoperative imaging may not be performed, as was the case with the patient in this report. A prospective cohort study from 1998 established that the gold standard for diagnosing hernias is through a clinical examination, with a sensitivity of 0.745 and specificity of 0.963. Further diagnostic investigation is typically warranted when groin pain is obscure, potentially indicating an occult hernia [16, 17, 18, 19].

It is important to note that while imaging was considered in this case, the conflict in Sudan has severely impacted healthcare resources. Many patients, including our case subject, found it challenging to access imaging modalities such as computed tomography (CT) or magnetic resonance imaging (MRI). The financial burden of traveling to distant cities for imaging further complicated the diagnostic process, making it necessary for clinicians to rely predominantly on physical examinations. Fortunately, ultrasound became available about a month post‐surgery, confirming an umbilical hernia while ruling out the presence of other neurofibromas.

To minimize the risk of misdiagnosis in such cases, a comprehensive history and thorough physical examination are imperative. Clinicians should meticulously assess the characteristics of the mass, focusing on aspects such as mobility, consistency, and its relationship to surrounding skin and structures. If there remains any uncertainty, detailed imaging is warranted. On ultrasound, solitary neurofibromas are often described as well‐defined, hypoechoic, and non‐compressed masses [6, 20]. While ultrasound is sensitive for identifying such lesions, it lacks the requisite specificity for achieving a definitive diagnosis [21]. CT is invaluable for discerning bone involvement and evaluating the displacement of adjacent structures [22]. MRI serves a crucial role in assessing soft tissue features. Neurofibromas show high intensity on T2‐weighted images and heterogeneous enhancement following contrast on T1‐weighted images; they typically show the target sign [21, 22, 23, 24]. Additionally, 18‐fluorodeoxyglucose‐positron emission tomography/computed tomography (FDG PET/CT) holds promise for distinguishing benign neurofibromas from malignant peripheral nerve sheath tumors, with an emphasis on analyzing metabolic activity [25]. Moreover, studies indicated that FDG PET/CT has high sensitivity (0.99) but lower specificity (0.53) for detecting malignant transformation in neurofibromas [26].

A definitive diagnosis of neurofibroma is achieved through histological analysis. The analysis usually reveals a composition of Schwann cells, fibroblasts, and collagen, often arranged within a myxoid matrix. The immunohistochemical profile of neurofibromas typically shows positivity for S100 and CD34 [27, 28]. In this case, the microscopic findings revealed all elements of peripheral nerves, including Schwann cells, collagen fibrils, and neurofilaments, confirming the diagnosis of a neurofibroma. Luckily, there were no atypical cells or indications of malignancy. While immunohistochemical studies were unavailable, the characteristic histological features strongly supported the diagnosis.

For clarity, Table 1 compares neurofibroma with its differential diagnoses, including the key imaging and histopathological characteristics. Fortunately, benign tumors like neurofibromas generally respond favorably to marginal excision, and surgical intervention yields excellent cosmetic outcomes. Excision remains the primary treatment approach for solitary neurofibromas [29]. Minimally invasive surgical approaches, such as endoscopic surgery, were used successfully in removing neurofibromas in specific locations [30, 31]. For asymptomatic solitary neurofibromas that are not causing functional impairment or cosmetic concern, observation is a viable option. Regular monitoring with imaging studies, such as MRI, can help detect any changes in tumor size or characteristics over time [32, 33].

**TABLE 1:** Compares neurofibroma with its differential diagnoses, including the key imaging and histopathological characteristics.

Lesion	Nature	Imaging characteristics	Histopathological features
Neurofibroma	Benign peripheral nerve sheath tumor	CT shows a well‐defined, hypodense mass, with homogeneous intensity. MRI shows a target sign with high intensity on T2 and heterogeneity on T1.	Shows Schwann cells, myxoid matrix, and positivity for S100 and CD34.
Schwannoma	Benign tumor of Schwann cells.	CT and MRI are both similar to neurofibroma but may show more irregular borders.	Shows Antoni A and B areas.
Hernia	Protrusion of an organ through a defect in the surrounding tissue.	CT and MRI both can show the herniated contents and the defect in the abdominal wall.	Lacks a specific signature.
Lipoma	Benign tumor of adipose tissue.	CT shows a homogeneous, low‐density mass with a well‐defined margin. MRI shows a hyperintensity on T1.	Shows mature adipocytes.
Dermatofibroma	Benign fibrous tumor of the skin.	CT shows a small, well‐defined, hypodense lesion. MRI shows low T1 and high T2 signal.	Shows fibrohistiocytic proliferation with hyperpigmentation.
Vascular malformation	Abnormal blood vessel formation.	CT density depending on the vascularity. MRI shows highT2 with flow voids indicating vascularity.	Shows endothelial cells and varying degrees of smooth muscle.

In summary, clinicians should be cautious when encountering painless, slowly growing masses in the groin region, as these can easily be mistaken for hernias. It is important to consider a broad differential diagnosis, including neurofibromas, to avert misdiagnosis and ensure appropriate management. The precise global prevalence of solitary neurofibromas remains ambiguous due to limited epidemiological data, underscoring the necessity for further research to establish their prevalence within the general population. This case exemplifies the challenges faced in clinical practice, particularly in low‐resource settings, emphasizing the critical need for a systematic approach to ensure accurate diagnoses and optimal management for patients presenting with similar conditions.

## Author Contributions

**Alaa Tajeldeen Habeeb Abdallah:** conceptualization, investigation, supervision, validation, visualization, writing – original draft, writing – review and editing. **Zainab Hassan Nooreldayem:** conceptualization, investigation, writing – original draft. **Rami Elsiddig Abdelkhalig:** conceptualization, supervision, writing – review and editing. **Elwasila Hamid:** conceptualization, supervision, writing – original draft, writing – review and editing. **Nouh Saad Mohamed:** conceptualization, supervision, writing – review and editing. **Ayman Ahmed:** conceptualization, supervision, validation, writing – original draft, writing – review and editing. **Emmanuel Edwar Siddig:** conceptualization, supervision, validation, writing – original draft, writing – review and editing.

## Funding

The authors have nothing to report.

## Consent

Written informed consent was obtained from the patient to publish this report in accordance with the journal's patient consent policy.

## Conflicts of Interest

The authors declare no conflicts of interest.

## Data Availability

Data available on request from the authors.
